# Prognosis in HR-positive metastatic breast cancer with HER2-low versus HER2-zero treated with CDK4/6 inhibitor and endocrine therapy: a meta-analysis

**DOI:** 10.3389/fonc.2024.1413674

**Published:** 2024-08-29

**Authors:** Lin-Yu Xia, Xu-Chen Cao, Qing-Lin Hu, Wei-Yun Xu

**Affiliations:** ^1^ Department of Thyroid and Breast Surgery, Clinical Medical College and The First Affiliated Hospital of Chengdu Medical College, Chengdu, Sichuan, China; ^2^ The First Department of Breast Cancer, Tianjin Medical University Cancer Institute and Hospital, National Clinical Research Center for Cancer, Tianjin, China; ^3^ Department of Breast Surgery, Mianyang Central Hospital, Mianyang, Sichuan, China

**Keywords:** breast cancer, HER2-low, CDK4/6 inhibitor, endocrine therapy, prognosis

## Abstract

**Background:**

The combination of CDK4/6 inhibitors (CDK4/6i) and endocrine therapy (ET) is currently the standard first-line treatment for patients with metastatic hormone receptor positive (HR+), and HER2-negative (HER2-) breast cancer. However, the impact of HER2 status on the prognosis of patients receiving CDK4/6i and ET remains unclear. The meta-analysis was conducted to evaluate different outcomes between HER2-low and HER2-zero patients in advanced HR+ breast cancer receiving CDK4/6i and ET.

**Methods:**

A systematic search was performed in PubMed and EMBASE databases for relevant published literature. Objective response rate (ORR), overall survival (OS), and progression-free survival (PFS) were pooled by fixed or random effects models.

**Results:**

Overall, 12 studies with 3567 patients were eligible for analysis. The pooled analysis suggested that no significant differences were observed in terms of ORR and OS between HER2-low and HER2-zero patients who underwent CDK4/6i and ET. Similarly, no significant difference in PFS was found between HER2-low and HER2-zero patients who underwent post-line CDK4/6i and ET or first-line Palbociclib and ET. However, in patients who received mixed-line (not a single treatment line) or first-line CDK4/6i and ET, the PFS was significantly shorter in the HER2-low subgroup than in the HER2-zero subgroup (mixed-line: HR = 1.36; 95% CI = 1.11–1.65; *P* = 0.002; first-line: HR = 1.14; 95% CI = 1.01–1.28; *P* = 0.04). A similar phenomenon was observed in patients who received mixed-line or post-line Palbociclib and ET (mixed-line: HR = 1.60; 95% CI = 1.09–2.34; *P* = 0.02; post-line: HR = 1.43; 95% CI = 1.03–2.00; *P* = 0.03).

**Conclusion:**

These results indicated that HER2-low status did not have a significant association with ORR and OS, but it may have a worse impact on PFS in patients who received mixed-line or first-line CDK4/6i and ET, as well as mixed-line or post-line palbociclib plus ET.

## Introduction

In recent years, new antibody-drug conjugate (ADC) drugs have been continuously developed. Destiny-Breast 04 has proved that new ADC are not only effective in HER2-positive breast cancer but also have good anti-tumor effects in HER2-low breast cancer (1, 2). This led to the concept of HER2-low breast cancer and aroused the research interest of scholars. Currently, HER2-low breast cancer is defined as tumors with an immunohistochemical (IHC) score of 1+ or 2+ and negative *in situ* hybridization (ISH) results, accounting for approximately 45%-55% of all breast cancers (3, 4).

CDK4/6i combined with ET can significantly improve the prognosis of patients with HR+/HER2- metastatic breast cancer (MBC) and has become the standard treatment for such patients (5–7). Previous studies have shown bidirectional crosstalk between ER and HER2 pathways. HER2 over-expression can reduce ER expression, regulate ER transcriptional activity, and cause ET resistance and drug resistance (8, 9). It is unclear whether HER2-low could cause endocrine resistance and drug resistance. Recent evidence shows that the expression of HER2-low is significantly higher in HR+ breast cancer compared with HR-negative breast cancer (10, 11). Moreover, Several studies have shown that HER2-low breast cancer has unique clinical biological characteristics. Compared with HER2-zero breast cancer, it has a lower Ki-67 score, lower histological grade, and less axillary lymph node metastasis (12–14). Therefore, it is particularly important to know whether HER2-low affects the prognosis of patients receiving CDK4/6i combined with ET. Some studies believe that HER2-low does not affect the survival outcomes of MBC patients treated with CDK4/6i plus ET (15–18). Other studies have suggested that HER2-low is associated with worse survival outcomes in MBC patients treated with CDK4/6i and ET (19–22). Given the conflicting results, we conducted this study to evaluate whether outcomes differ between HR+ MBC patients with HER2-low and HER2-zero treated with CDK4/6i and ET.

## Materials and methods

### Search strategy

The meta-analysis was conducted according to the PRISMA statement (23). The PRISMA checklist is provided in **Supplementary Table 1**. A systematic search was conducted in the PubMed and Embase databases for studies published by January 2024. It was performed using the following keywords: “HER2-low” OR “ERBB2 low” AND “CDK 4/6” OR “Palbociclib” OR “Ribociclib” OR “Abemaciclib”. The details of the search strategy are provided in **Supplementary Table 2**. The study was registered in PROSPERO (CRD42024521829).

### Inclusion and exclusion criteria

Publications were considered eligible if they met all the following inclusion criteria; 1) patients diagnosed with HR+/HER2- MBC; 2) patients who received CDK4/6i plus ET (aromatase inhibitor or fulvestrant); 3) the study compared the survival outcomes in terms of OS and/or PFS between HER2-low and HER2-zero. Exclusion criteria were as follows; 1) review articles, meta-analysis, case reports or letters; 2) patients with other malignant tumors; 3) HRs and 95% CIs cannot be extracted.

### Data extraction and quality assessment

Data were collected from the eligible studies independently by two investigators. The relevant information was extracted such as the first author’s name, nationality, publication year, study type, the number of participants, median age, median follow-up time, treatment line, metastatic sites type, CDK4/6i, treatment manner, ORR, OS, PFS.

The non-randomized experimental research-MINORS scale was used to assess the study quality of the included studies (24). Eight items needed to be evaluated. Each item was scored as 0, 1, or 2, corresponding to not reported, inadequately reported, or adequately reported. Studies with scores of 8 or more were rated as high-quality (See **Supplementary Table 3**).

### Summary measures and statistical analysis

The Meta-analysis was performed using RevMan version 5.3 (RevMan, version 5.3 for Windows; Cochrane Collaboration, Oxford, UK). The hazard ratios (HRs) and 95% CIs were directly extracted from reported data or calculated from Kaplan-Meier survival curves. Statistical heterogeneity within studies was detected with Chi-squared and I^2^. When I^2^ > 50%, the heterogeneity was significant and the random-effects model was used; otherwise, there was no heterogeneity and the fixed-effects model was used (25). Funnel plot and Begg’s test were performed with the Stata 11.0 (Stata Corporation, College Station, TX, USA) to assess the potential publication bias (26). All tests were two-sided. *P *< 0.05 was considered statistically significant.

## Results

### Study selection and characteristics

**Figure 1:**
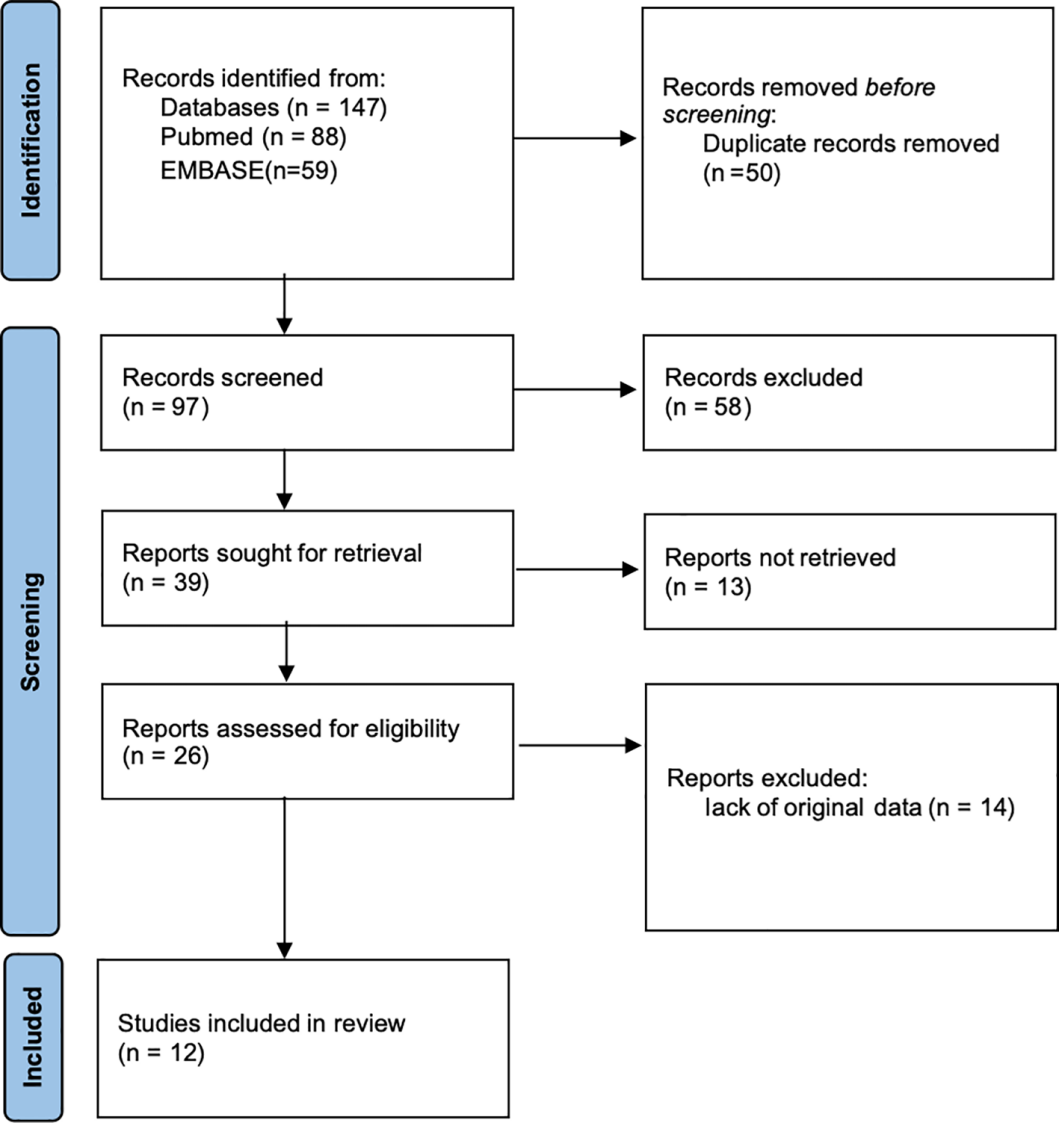
Flowchart explaining the article selection.

147 articles were identified from the two databases, of which 50 were duplications. After screening the titles and abstracts of the remaining 97 studies, 58 articles were removed. Another 27 articles were excluded when the full texts were examined. Finally, 12 studies with 3567 patients met the qualifying criteria were included (15–22, 27–30). **Figure 1** shows the flowchart of the literature selection.

All the studies were retrospective studies from 8 countries such as Italy, Argentina, China, Greece, and so on. The median age of patients ranges from 48.3 to 63. Median follow-up time ranges from 15 to 36. The follow up time was adequate for most of the studies. 11 studies reported the number of patients with HER2-low and HER2-zero respectively. Among 3359 patients, there were 1903 (56.65%) HER2-low patients and 1456 (43.35%) HER2-zero patients. In terms of CDK4/6i, Palbociclib was the most commonly used among patients included in the study. In 5 studies, patients were treated with CDK4/6i and ET as first-line treatment, while the remaining 7 studies also used it as second-line or third-line treatment. **Tables 1**, **2** show the study characteristics.

**Table 1:** Main characteristics of the eligible studies.

First author	year	country	study type	participations(n)	median age	CDK4/6i	line of treatment	Follow-up (median) (month)	outcomes
Bortot	2021	Italy	retrospective	84	NR	NR	first	NR	OS、PFS
Bao	2021	China	retrospective	106	58	Palbo	mixed	NR	PFS
Lapuchesky	2022	Argentina	retrospective	186	55	Palbo+Ribo+Abe	mixed	NR	OS、PFS
Shao	2022	China	retrospective	45	50	Palbo	mixed	NR	ORR、PFS
Douganiotis	2022	Greece	retrospective	189	60	Palbo+Ribo	first	15	PFS
Carlino	2022	Italy	retrospective	165	64	Palbo	first	31	OS、PFS
Yildirim	2023	Turkey	retrospective	204	58	Palbo+Ribo	mixed, first, post	22	ORR、PFS
Zattarin	2023	Italy	retrospective	428	NR	Palbo+Ribo+Abe	first	36	OS、PFS
Sharaf	2023	Jordan	retrospective	257	49.9	Ribo	mixed	NR	ORR、PFS
You	2023	China	retrospective	458	55	Palbo	first	NR	PFS
Mouabbi	2023	USA	retrospective	1084	50	Palbo+Ribo+Abe	mixed, first, post	17.9	OS、PFS
Liang	2024	China	retrospective	361	58	Palbo	mixed, first, post	29	OS、PFS

NR, Not reported; Palbo, Palbociclib; Ribo, Ribociclib; Abe, heluuAbemaciclib.

**Table 2:** Patient and treatment characteristics of the the eligible studies.

First author	participations	treatment line		treatment manner		CDK4/6 inhibitor	
	HER2-low	HER2-low	HER2-zero	CDK4/6i + AI/CDK4/6i + Ful		HER2-low	HER2-zero
	/HER2-zero	First/post	First/post	HER2-low	HER2-zero	Palbo/Ribo/Abe	Palbo/Ribo/Abe
Bortot	NR	NR	NR	NR	NR	NR	NR
Bao	82/24	39/21	15/6	NR	NR	68/14/0	22/2/0
Lapuchesky	64/122	NR	NR	NR	NR	NR	NR
Shao	21/24	7/14	7/17	12/9	12/12	21/0/0	24/0/0
Douganiotis	137/52	137/0	52/0	NR	NR	NR	NR
Carlino	71/94	71/0	94/0	NR	NR	71/0/0	94/0/0
Yildirim	66/138	25/41	74/64	32/34	83/55	26/40/0	53/85/0
Zattarin	269/159	NR	NR	161/108	94/65	186/55/28	105/36/18
Sharaf	143/114	84/59	78/36	NR	NR	0/143/0	0/114/0
You	249/209	24/25	19/27	21/28	24/22	49/0/0	46/0/0
Mouabbi	697/387	587/110	325/62	NR	NR	NR	NR
Liang	228/133	NR	NR	NR	NR	NR	NR

Ful, Fulvestrant; Palbo, Palbociclib; Ribo, Ribociclib; Abe, Abemaciclib; NR, Not reported.

### Objective response rate

**Figure 2:**
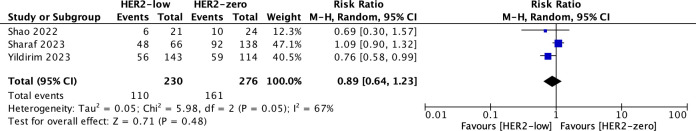
Forest plot of the RR for the ORR of HER2-low breast cancer vs. HER2-zero breast cancer.

3 studies with 506 patients reported ORR. The ORR was 47.83%(110/230) in patients with HER2-low and 58.33%(161/276) in patients with HER2-zero. There was no significant difference in ORR between HER2-low and HER2-zero patients (HR = 0.89; 95% CI = 0.64–1.23; *P* = 0.48). Heterogeneity was not detected among the included studies (I^2^ = 67%, *P* = 0.05). As shown in **Figure 2**.

### Overall survival

**Figure 3:**
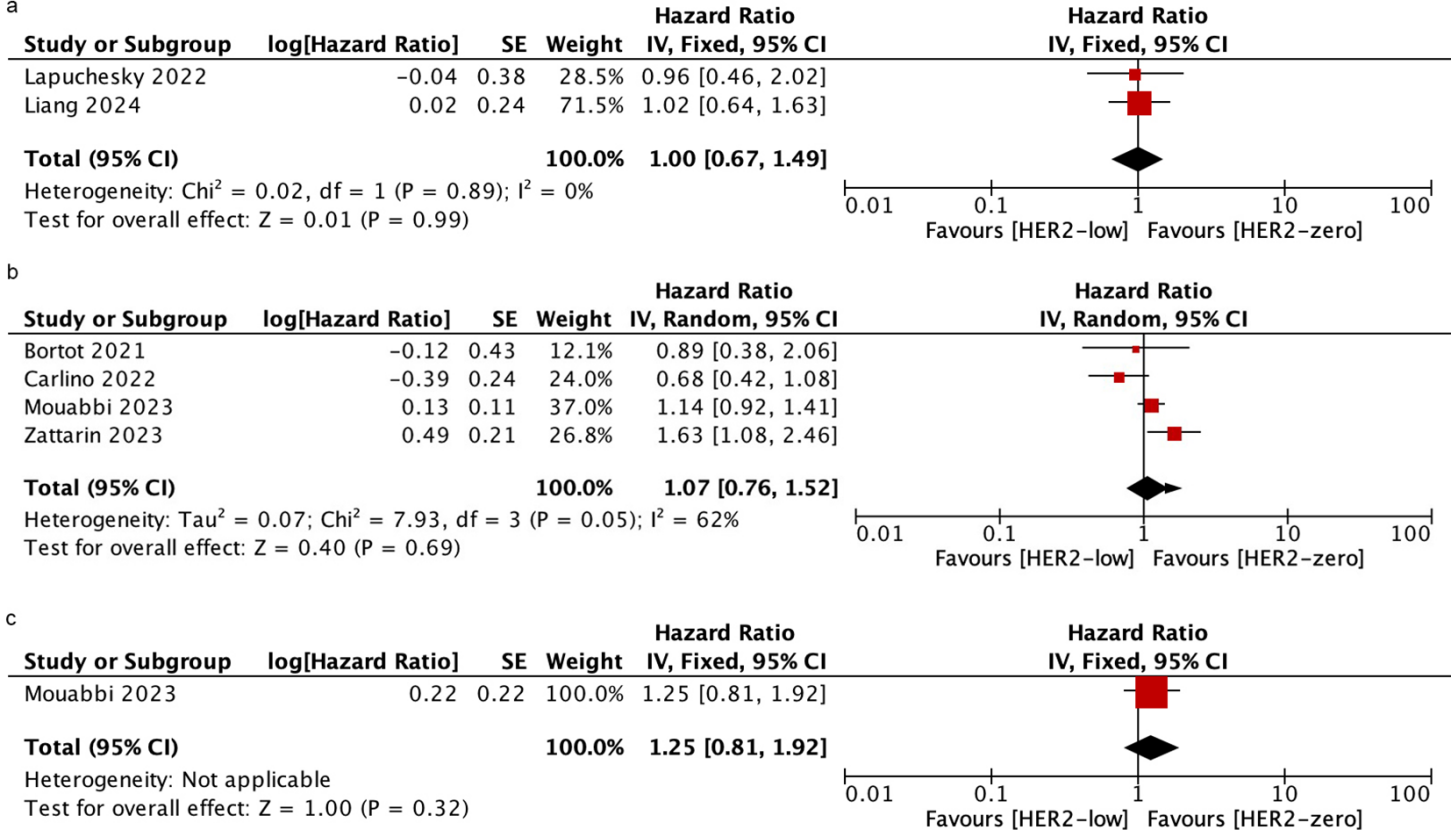
Forest plot of the HR for OS of HER2-low breast cancer vs. HER2-zero breast cancer in mixed-line **(A)**, first-line **(B)**, post-line **(C)**.

Six studies involving 2306 patients reported OS. The OS of HER2-low VS. HER2-zero in patients who received CDK4/6i plus ET as mixed-line therapy was evaluated in the Lapuchesky and Liang trials. The results revealed there was no statistically significant difference in OS between the two groups (HR = 1.00; 95% CI = 0.67–1.49; *P* = 0.99). 4 studies reported the OS in patients who were treated with first-line and only one study reported those treated with post-line. The OS of HER2-low VS. HER2-zero patients was similar in both of the two subgroups (first-line: HR = 1.07; 95% CI = 0.76–1.52; *P* = 0.69; post-line: HR = 1.25; 95% CI = 0.81–1.92; *P* = 0.32). No heterogeneity was detected within the included studies (mixed-line: I^2^ = 0%, *P* = 0.89; first-line: I^2^ = 62%*, P* = 0.05). As shown in **Figure 3**.

### Progression free survival

**Figure 4:**
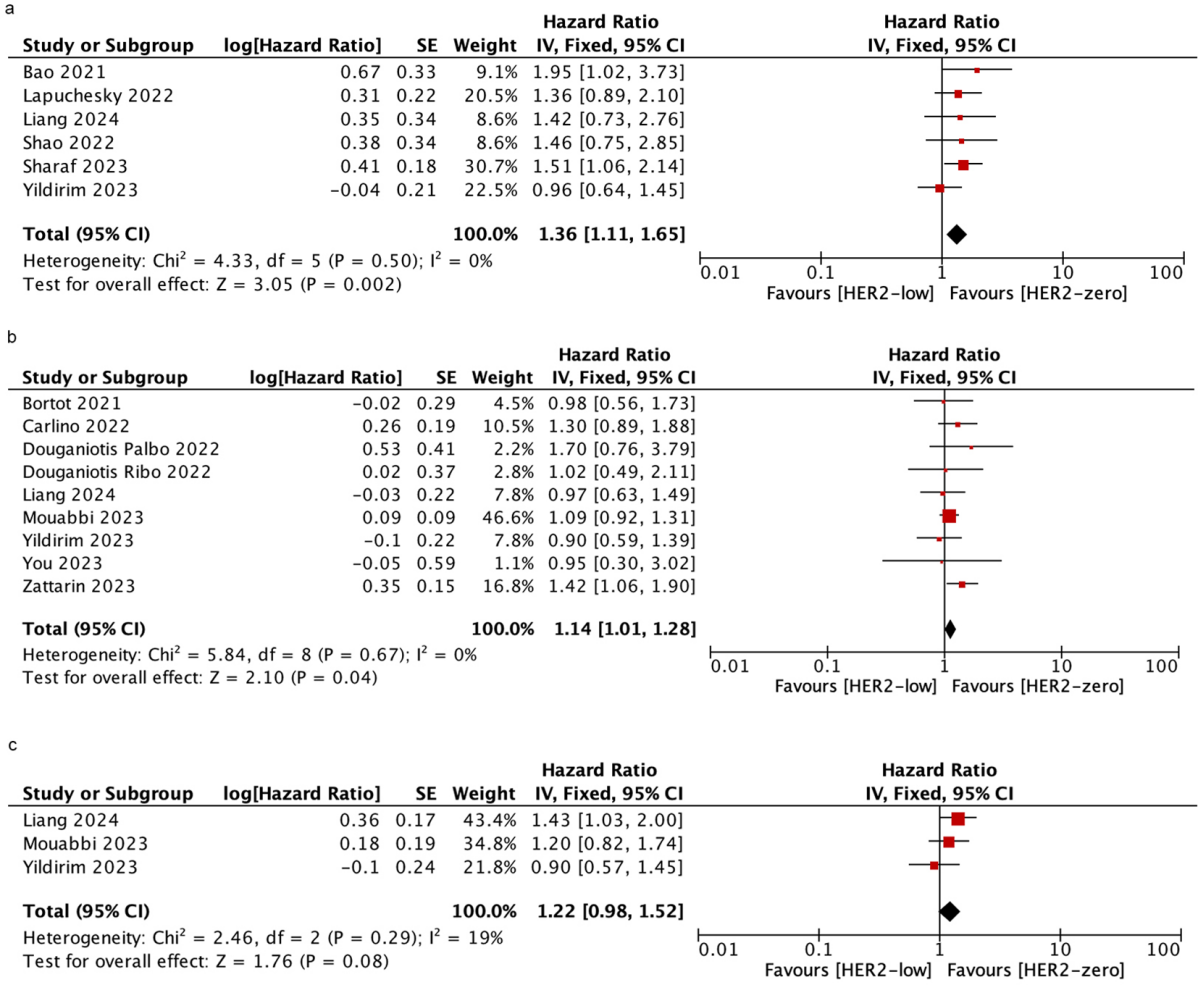
Forest plot of the HR for PFS of HER2-low breast cancer vs. HER2-zero breast cancer in mixed-line **(A)**, first-line **(B)**, post-line **(C)**.

Six studies were included to assess the PFS in patients who received mixed-line treatment. The results revealed that the PFS was shorter in the HER2-low subgroup than in the HER2-zero subgroup (HR = 1.36; 95% CI = 1.11–1.65; *P* = 0.002). Similarly, in patients who were treated with first-line, HER2-low patients had shorter PFS than HER2-zero patients based on nine studies (HR = 1.14; 95% CI = 1.01–1.28; *P* = 0.04). However, data obtained from three studies demonstrated there was no significant difference in PFS between HER2-low and HER2-zero patients who were treated with post-line (HR = 1.22; 95% CI = 0.98–1.52; *P* = 0.08). There was no significant heterogeneity among the studies. As shown in **Figure 4**.

**Figure 5:**
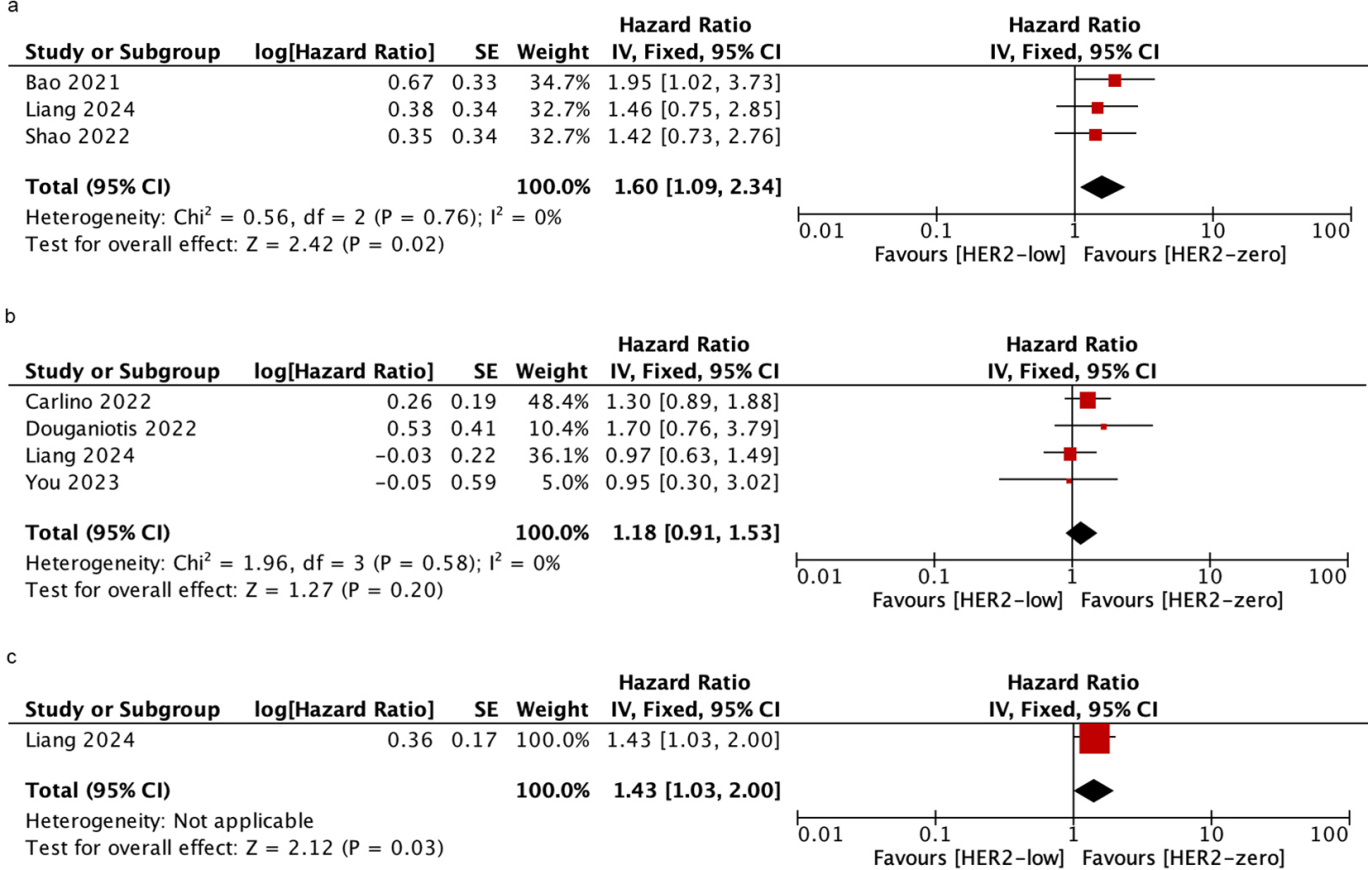
Forest plot of the HR for PFS (palbociclib plus ET) of HER2-low breast cancer vs. HER2-zero breast cancer in mixed-line **(A)**, first-line **(B)**, post-line **(C)**.

Palbociclib is the most commonly used CDK4/6i in the included studies. To determine whether HER2-low was associated with PFS in patients who were treated with palbociclib plus ET, further analyses were performed. we assessed three studies to evaluate the PFS in patients who received mixed-line treatment. The results showed that HER2-low patients had shorter PFS than HER2-zero patients (HR = 1.60; 95% CI = 1.09–2.34; *P* = 0.02). Furthermore, 4 studies and 1 study were included to assess the PFS in patients who received first-line treatment and post-line treatment, respectively. There was no significant difference in PFS between HER2-low and HER2-zero patients during the first-line treatment (HR = 1.18; 95% CI = 0.91–1.53; *P* = 0.2), while a significantly poor PFS was observed in HER2-low patients compared to those with HER2-zero in the post-line treatment subgroup (HR = 1.43; 95% CI = 1.03–2.00; *P* = 0.03). No heterogeneity was detected within the included studies. As shown in **Figure 5**.

**Figure 6:**
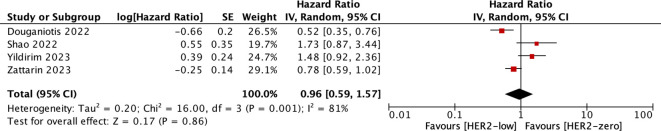
Forest plot of the HR for PFS of CDK4/6i+AIs vs. CDK4/6i+fulvestrant.

Four studies included in our research compared the PFS in patients treated with CDK4/6i+AIs versus CDK4/6i+fulvestrant. We conducted a summary analysis, concluding that there was no difference in PFS between the patients who received CDK4/6i+AIs versus CDK4/6i+fulvestrant (HR = 0.96; 95% CI = 0.59–1.57; *P* = 0.86). As shown in **Figure 6**.

The funnel plots and Begg’s test were conducted to evaluate the publication bias (See **Supplementary Figure 1**). All *P* values were > 0.05, which revealed there was no significant publication bias found in ORR, OS, and PFS (See **Supplementary Table 4**).

## Discussion

CDK4/6i and ET are the standard treatments for the vast majority of HR+/HER2- MBC patients. At present, studies on the effect of HER2-low on treatment outcomes in patients with HR+ MBC receiving CDK 4/6i + ET are quite limited and the results are contradictory. In this study, we investigated the association between HER2 status (low vs. zero) and prognosis in HR+/HER2- MBC patients treated with CDK4/6i and ET through a pooled analysis of 12 studies. The results showed that HER2-low was not associated with ORR and OS, yet it was significantly linked to worse PFS in certain treatment lines. In addition, Douganiotis et al. also pointed out that “Median PFS was numerically different among the different expression level of Her2(0 VS.1+, 0 VS. 2+), but this difference did not reach statistical significance (*P*=0.477) (28).

New antibody-targeting drugs have drawn our attention to HER2-low breast cancer. More and more studies have begun to compare the difference between HER2-zero breast cancer and HER2-low breast cancer. Studies have proven that histological grade 2 tumors are more common in HER2-low breast cancer patients, and the expression level of Ki67 is significantly lower than that in HER2-zero patients (13, 31). In terms of gene expression, compared with HER2-zero, HER2-low breast cancer has fewer TP53 mutations, proliferation-related genes are significantly down-regulated, and lumen-related genes are significantly up-regulated (11, 32). Therefore, scholars speculate that HER2-low is related to a good prognosis. However, studies have produced conflicting results. Many studies have reported that the prognosis of HER2-low breast cancer is better than that of HER2-zero breast cancer (33–35), but some studies have reported that the prognosis of the two groups is the same (36–38). Conflicting results were also obtained on the efficacy of neoadjuvant chemotherapy in the two groups. Some studies have shown that HER2-low is associated with lower pathological complete response (pCR) rates (39–41). Another part of the study showed that there was no significant difference in pCR rate between the two groups (42–44). Therefore, HER2-low as a unique biological subtype of breast cancer is still controversial.

Resistance to ET often occurs, and studies have shown that this is related to the over-activity of the RTK signaling pathway, in which overexpressed HER2 plays an important role (45). The HER family comprises 4 RTKs (HER1-4) and several of their ligands (46). The activation of HER2 can induce the transphosphorylation of ERBB dimer partners and stimulate multiple intracellular pathways, such as RAS/RAF/MEK/ERK, PI3K/AKT/TOR, Src kinase, and STAT transcription factors. The activation of these pathways can significantly interfere with ER transcriptional activity, which may lead to endocrine resistance (47). Recent studies have also demonstrated that members of the HER signaling pathway can reduce ER expression at both mRNA and protein levels, leading to decreased endocrine sensitivity (48). Several studies have shown that the estrogen receptor positive (ER+) breast cancer with HER2 over-expression shows resistance to ET (49–51). Compared with simple ET, the addition of anti-HER2 treatment into ET can also significantly improve the efficacy of HR+/HER2+ advanced breast cancer patients (52). It is unclear whether HER2-low expression contributes to resistance to ET. Wang et al. analyzed the prognosis of 72 HR+/HER2- advanced breast cancer patients who received first-line ET, and the study showed that the OS and PFS of HER2-low patients were lower than those of HER2-zero patients (53). Wu et al. analyzed 233 women with HR+/HER2- MBC who received ET with or without CDK4/6 inhibitors (54). The results showed that HER2-low subgroup showed a significantly shorter median PFS compared to the HER2-zero subgroup in the ET alone cohort (5.6 VS. 17.0 months; *P* = 0.0044). These seem to indicate that HER2-low patients are more likely to develop endocrine resistance. Collins et al. conducted a study on the ER+/HER2-low human breast cancer mouse xenotransplantation model, and the results showed that adding ET to the combination of anti-HER3 and anti-HER2 drugs could further improve the efficacy of tumor regression (55). In addition, in patients with ER+/HER2-low breast cancer who failed ET, the combination of lumretuzumab and pertuzumab treatment could prolong clinical response. These results confirm that there is also direct crosstalk between HER2 and ER in HER2-low breast cancer.

HR+/HER2- advanced breast cancer patients have poor chemo-sensitivity and poor prognosis after endocrine resistance. The development of CDK4/6i has brought new hope to such patients. Several large clinical studies have shown that compared with ET treatment alone, combined with CDK4/6i can achieve a higher ORR for patients and can significantly improve the PFS and OS of patients. Therefore, several authoritative guidelines unanimously recommend that CDK4/6i combined with ET is currently the standard treatment for patients with HR+/HER2- MBC (56–58). However, in clinical practice, there are still people who progress rapidly after first-line CDK4/6i + ET treatment, and the treatment effect is limited. There are currently no clear molecular markers for predicting therapeutic effects. Our research shows that HER2-low may have a worse impact on PFS in patients who received CDK4/6i and ET. Given this, we may need to improve the prognosis of HR+/HER2-low breast cancer patients who receive CDK4/6i and ET. Nearly 70% of HR+/HER2-low breast cancer patients in the DESTINY-Breast 04 study had previously received CDK4/6i treatment. Results showed that among these patients, the mPFS of the trastuzumab deruxtecan (T-DXd) group was twice that of the control chemotherapy group, significantly reducing the risk of disease progression or death (1). Based on this, we speculate whether CDK4/6i +T-DXd treatment can improve the prognosis of HR+/HER2-low patients, but there is currently no relevant research. The 2023 SABCS conference updated the results of the DESTINY-Breast 08 study. The ORR of the T-DXd combined with anastrozole group can reach 70%, and the median PFS reaches 13.4 months; the ORR of T-DXd combined with fulvestrant is 40%, and the median PFS was not reached. Studies have shown that the combination of T-DXd with anastrozole or fulvestrant has positive significance in the first or second-line treatment of HER2-low expression HR+ MBC patients (59). We look forward to future studies on T-DXd combined with CDK4/6i.

Our meta-analysis has a few limitations. Firstly, all the included studies are retrospective, which may have heterogenous in terms of menopausal status, levels of ER/PgR, analysis of HER2 on primary or metastases, intra- and inter-heterogeneity. Secondly, some subgroup analyses only included a few studies, We should interpret their results with caution. Thirdly, there are some confounding factors such as different CDK4/6i and different hormonal therapy companions (AIs and fulvestrant) were used in these studies. We were not able to perform an analysis by different CDK4/6i because no data was available for that. Finally, there may be some differences between the actual HR and that extracted from the survival curve, which can affect the final result we calculate.

## Conclusion

In conclusion, our meta-analysis suggested that HR+ MBC patients with HER2-low and HER2-zero have similar ORR and OS when receiving CDK4/6i and ET treatment, but HER2-low may have a worse impact on PFS in patients who received mixed-line or first-line CDK4/6i and ET, as well as mixed-line or post-line palbociclib plus ET. Further large prospective studies are needed to confirm this result.

## Data Availability

The original contributions presented in the study are included in the article/**Supplementary Material**. Further inquiries can be directed to the corresponding author.

## Supplementary material

Supplementary Figure 1 Funnel plot of HER2-low breast cancer vs. HER2-zero breast cancer. RR for the ORR **(A)**; HR for OS: mixed-line **(B)**, first-line **(C)**; HR for PFS: mixed-line **(D)**, first-line **(E)**, post-line **(F)**; HR for PFS (palbociclib plus ET): mixed-line **(G)**, first-line **(H)**; HR for PFS (CDK4/6i+AIs vs. CDK4/6i+fulvestrant) **(I)**
