# Supplementary material for: Prognosis in HR-positive metastatic breast cancer with HER2-low versus HER2-zero treated with CDK4/6 inhibitor and endocrine therapy: a meta-analysis
Source: Front Oncol. 2024 Aug 29;14:1413674. doi: 10.3389/fonc.2024.1413674 (PMC11390584; doi:10.3389/fonc.2024.1413674)
Supplement: Supplementary file 2 [file Table2.doc]

| Supplementary Table 2. Detailed search strategy | |
| --- | --- |
| Database | Search strategy |
| Pubmed | (((((((breast cancer) OR (breast tumor)) OR (breast neoplasm)) OR (breast carcinoma))) AND ((((HER2 low) OR (ERBB2 low)) OR (low HER2)) OR (low ERBB2))) AND ((((CDK 4/6) OR (Palbociclib)) OR (Ribociclib)) OR (Abemaciclib)) |
| Embase | 1.'breast cancer' OR 'breast tumor' OR 'breast neoplasm' OR 'breast carcinoma'  2.'HER2 low' OR 'ERBB2 low' OR 'low HER2' OR 'low ERBB2'  3.'CDK 4/6' OR 'Palbociclib' OR 'Ribociclib' OR 'Abemaciclib'  4. 1 and 2 and 3 |
|  | |
