# Supplementary material for: Prognosis in HR-positive metastatic breast cancer with HER2-low versus HER2-zero treated with CDK4/6 inhibitor and endocrine therapy: a meta-analysis
Source: Front Oncol. 2024 Aug 29;14:1413674. doi: 10.3389/fonc.2024.1413674 (PMC11390584; doi:10.3389/fonc.2024.1413674)
Supplement: Supplementary file 3 [file Table3.docx]

Supplementary Table 3. Risk of bias in the included cohort studies (by the MINORS quality assessment tool)

|  | **A clearly stated aim** | **Inclusion of consecutive patients** | **Prospective collection of data** | **Endpoints appropriate to the aim of the study** | **Unbiased assessment of the study endpoint** | **Follow-up period appropriate to the aim of the study** | **Loss to follow up less than 5%** | **Prospective calculation of the study size** | **Total quality scores** |  |
| --- | --- | --- | --- | --- | --- | --- | --- | --- | --- | --- |
| Bortot | 2 | 2 | 0 | 2 | 2 | 2 | 2 | 0 | 12 |  |
| Lapuchesky | 2 | 2 | 0 | 2 | 2 | 2 | 2 | 0 | 12 |  |
| Bao | 2 | 2 | 0 | 2 | 2 | 2 | 2 | 0 | 12 |  |
| Shao | 2 | 2 | 0 | 2 | 1 | 1 | 2 | 0 | 10 |  |
| Douganiotis | 2 | 2 | 0 | 2 | 2 | 1 | 2 | 0 | 11 |  |
| Carlino | 2 | 2 | 0 | 2 | 2 | 2 | 2 | 0 | 12 |  |
| Yildirim | 2 | 2 | 0 | 2 | 2 | 2 | 2 | 0 | 12 |  |
| Zattarin | 2 | 2 | 0 | 2 | 2 | 2 | 2 | 0 | 12 |  |
| Sharaf | 2 | 2 | 0 | 2 | 2 | 2 | 2 | 0 | 12 |  |
| You | 2 | 2 | 0 | 2 | 2 | 2 | 2 | 0 | 12 |  |
| Mouabbi | 2 | 2 | 0 | 2 | 2 | 1 | 2 | 0 | 11 |  |
| Liang | 2 | 2 | 0 | 2 | 2 | 2 | 2 | 0 | 12 |  |
