# Supplementary material for: Prognosis in HR-positive metastatic breast cancer with HER2-low versus HER2-zero treated with CDK4/6 inhibitor and endocrine therapy: a meta-analysis
Source: Front Oncol. 2024 Aug 29;14:1413674. doi: 10.3389/fonc.2024.1413674 (PMC11390584; doi:10.3389/fonc.2024.1413674)
Supplement: Supplementary file 4 [file Table4.docx]

Supplementary Table 4 Results of Begg’s tests for publication bias

| End point | z | *P* |
| --- | --- | --- |
| ORR | 0 | 1 |
| OS: mixed-line (HER2-low VS. HER2-zero) | 0 | 1 |
| OS: first-line (HER2-low VS. HER2-zero) | 0.34 | 0.734 |
| PFS: mixed-line (HER2-low VS. HER2-zero) | 0.19 | 0.848 |
| PFS: first-line (HER2-low VS. HER2-zero) | 0.21 | 0.834 |
| PFS: post-line (HER2-low VS. HER2-zero) | 1.04 | 0.296 |
| PFS: Palbo mixed-line (HER2-low VS. HER2-zero) | 0.61 | 0.540 |
| PFS: Palbo first-line (HER2-low VS. HER2-zero) | -0.34 | 1 |
| PFS: AI VS. FUL | 0.34 | 0.734 |
