# Supplementary figures and images for: Prognosis in HR-positive metastatic breast cancer with HER2-low versus HER2-zero treated with CDK4/6 inhibitor and endocrine therapy: a meta-analysis
Source: Front Oncol. 2024 Aug 29;14:1413674. doi: 10.3389/fonc.2024.1413674 (PMC11390584; doi:10.3389/fonc.2024.1413674)

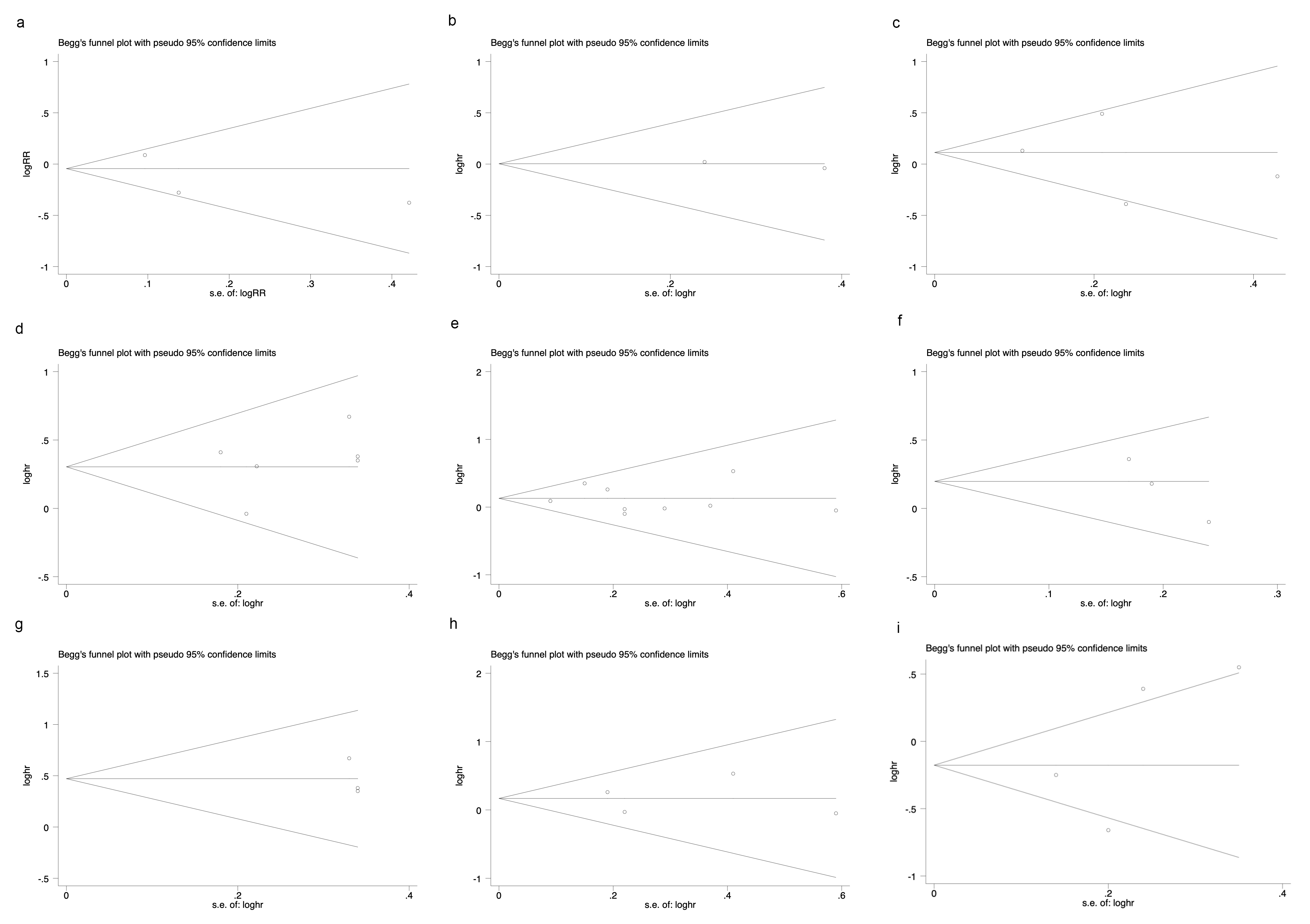

Supplement: Supplementary Figure 1 — Funnel plot of HER2-low breast cancer vs. HER2-zero breast cancer. RR for the ORR (A); HR for OS: mixed-line (B), first-line (C); HR for PFS: mixed-line (D), first-line (E), post-line (F); HR for PFS (palbociclib plus ET): mixed-line (G), first-line (H); HR for PFS (CDK4/6i+AIs vs. CDK4/6i+fulvestrant) (I) [file Image1.jpeg]
